# Gel-Based and Gel-Free Quantitative Proteomics Approaches at a Glance

**DOI:** 10.1155/2012/494572

**Published:** 2012-11-20

**Authors:** Cosette Abdallah, Eliane Dumas-Gaudot, Jenny Renaut, Kjell Sergeant

**Affiliations:** ^1^Environment and Agro-Biotechnologies Department, Centre de Recherche Public-Gabriel Lippmann, 41 rue du Brill, 4422 Belvaux, Luxembourg; ^2^UMR Agroécologie INRA 1347/Agrosup/Université de Bourgogne, Pôle Interactions Plantes Microorganismes ERL 6300 CNRS, Boite Postal 86510, 21065 Dijon Cedex, France

## Abstract

Two-dimensional gel electrophoresis (2-DE) is widely applied and remains the method of choice in proteomics; however, pervasive 2-DE-related concerns undermine its prospects as a dominant separation technique in proteome research. Consequently, the state-of-the-art shotgun techniques are slowly taking over and utilising the rapid expansion and advancement of mass spectrometry (MS) to provide a new toolbox of gel-free quantitative techniques. When coupled to MS, the shotgun proteomic pipeline can fuel new routes in sensitive and high-throughput profiling of proteins, leading to a high accuracy in quantification. Although label-based approaches, either chemical or metabolic, gained popularity in quantitative proteomics because of the multiplexing capacity, these approaches are not without drawbacks. The burgeoning label-free methods are tag independent and suitable for all kinds of samples. The challenges in quantitative proteomics are more prominent in plants due to difficulties in protein extraction, some protein abundance in green tissue, and the absence of well-annotated and completed genome sequences. The goal of this perspective assay is to present the balance between the strengths and weaknesses of the available gel-based and -free methods and their application to plants. The latest trends in peptide fractionation amenable to MS analysis are as well discussed.

## 1. Introduction

*“In the wonderland of complete sequences, there is much that genomics cannot do, and so the future belongs to proteomics, the analysis of complete complements of proteins” [1]*

Originally coined by Wilkins et al. in 1996, proteomics by name is now over 15 years old. The term “proteome” refers to the entire PROTEin complement expressed by a genOME [2]. Proteomics is thus the large-scale analysis of proteins in a cell, tissue, or whole organism at a given time under defined conditions. The cutting-edge proteomics techniques offer several advantages over genome-based technologies as they directly deal with the functional molecules rather than genetic code or mRNA abundance. Even though there is only one definitive genome of an organism, it codes for multiple proteomes since the accumulation of a protein changes in relation to the environment and is the result of a combination of transcription, translation, protein turnover, and posttranslational modifications. 

The field of proteomics has grown at an astonishing rate, mainly due to tremendous improvements in the accuracy, sensitivity, speed and throughput of mass spectrometry (MS), and the development of powerful analytical software. It appears to be gaining momentum as proteomic techniques become increasingly widespread and applied to an expanding smorgasbord of biological assays. Recently, proteomics has expanded from mere protein profiling to accurate and high-throughput protein quantification between two or multiple biological samples. 

Most of the early developments in quantitative proteomics were driven by research on yeast and mammalian cell lines [3]. The incidence of proteomic studies on plants has increased over the past years but still lags behind human and animal proteomics, moreover model organisms and cash crops (e.g., *Arabidopsis* and rice) continue to be dominant in the plant proteomic literature. Most quantitative proteomic techniques used for human, animal, or other eukaryotic organisms can essentially also be employed for plant systems but plants, possessing distinct properties with regard to their genome, physiology, and culture, can impose high demands on proteomic sample handling. However, these advanced strategies have helped and facilitated the study of plant proteins and many new reports on differential expression, as well as global and organellar proteomic elucidation, have been put forth. 

Quantitative proteomic approaches can be classified as either gel-based or gel-free methods as well as “label-free” or “label-based,” of which the latter can be further subdivided into the various types of labelling approaches such as chemical and metabolic labelling. In the present work, the thorough description and current status of commonly used gel-based and -free proteomic methodologies is provided. An overview of their suitability, potential, and bottleneck applications in plant proteomics is discussed. 

## 2. Gel-Based Proteomics

*“Electrophoresis today and tomorrow: helping biologist's dreams come true” [4]*

### 2.1. Two-Dimensional Gel Electrophoresis (2-DE): The Workhorse of Proteomics

Since it was first introduced in 1975 [5], 2-DE has evolved at different levels and became the workhorse of protein separation and the method of choice for differential protein expression analysis. Proteins first undergo isoelectric focusing (IEF) based on their net charge at different pH values and in the orthogonal second dimension further separation is performed based on the molecular weight (MW). This technique has an excellent resolving power, and today, it is possible to visualize over 10,000 spots corresponding to over 1,000 proteins, multiple spots containing different molecular forms of the same protein, on a single 2-DE gel [3]. Due to the pivotal problem of protein solubility, the overwhelming majority of electrophoretic protein separations is made under denaturing conditions. Two types of reagents are used in 2-DE buffers to ensure protein solubility and denaturation. The first type, chaotropes (e.g., urea and thiourea) used at multimolar concentrations, is able to unfold proteins by weakening noncovalent bonds (hydrophobic interactions, hydrogen bonds) between proteins [6]. The second one is ionic detergents, in which SDS (sodium dodecyl sulfate) is the archetype. It is made of a long and flexible hydrocarbon chain linked to an ionic polar head. The detergent molecules will bind through their hydrophobic hydrocarbon tail to hydrophobic amino acids. This binding favours amino acid-detergent interactions over amino acid-amino acid interactions, thereby promoting denaturation. Moreover, nonionic or zwitterionic detergents such as Triton X-100 are also used for protein solubilisation, since IEF requires low ion concentration in the sample [7]. The detection method postgel migration is achieved either by the use of visible stains such as silver and Coomassie or fluorescent stains such as Sypro Ruby, Lava and Deep Purple. 

Nevertheless, 2-DE has lately come under assault due to its known limitations and in part to the development of alternative MS-based approaches. Some of the reasons behind this trend include issues related to reproducibility [8], poor representation of low abundant proteins [9], highly acidic/basic proteins, or proteins with extreme size or hydrophobicity [10], and difficulties in automation of the gel-based techniques [11]. Moreover, the comigration of multiple proteins in a single spot renders comparative quantification rather inaccurate. 

Although no technique has a better resolving power than classical 2-DE, many endeavours were made to step forward and make it suitable to study membrane proteins [7], and to overcome the protein ratio errors due to low gel-to-gel reproducibility by the inclusion of difference gel electrophoresis (2D-DIGE) [12]. This technique enables protein detection at subpicomolar levels and relies on preelectrophoretic labelling of samples with one of three spectrally resolvable fluorescent CyDyes (Cy2, Cy3, and Cy5). These dyes have an NHS-ester reactive group that covalently attaches to the *ε*-amino group of protein lysines via an amide linkage. The ratio of dye to protein is specifically designed to ensure that the dyes are limiting in the reaction and approximately cover 1-2% of the available proteins where only a single lysine per protein is labelled. Intergel comparability is achieved by the use of an internal standard (mixture of all samples in the experiment) labelled with Cy2 and coresolved on the gels that each contains individual samples labelled with Cy3 or Cy5. Since every sample is multiplexed with an equal aliquot of the same Cy2 standard mixture, each resolved feature can be directly related to the Cy2-labelled internal standard, and ratios can be normalized to all other ratios from other samples and across different gels. This can be done with extremely low technical variability and high statistical power [13–15]. 

For quantitative analysis, imaging software is required to align gel spots and measure their intensities. To this end, gels need to be digitalised either by using a scanner recording light transmitted through or reflected from the stained gel or fluorescent scanner. The images are subsequently imported into dedicated commercially available 2-DE image analysis softwares such as DeCyder (GE Healthcare), Proteomweaver (Bio-Rad), PDQuest (Bio-Rad), and Progenesis Same Spots (Nonlinear Dynamics). Most of these analysis software tools are user-friendly and allow (i) image alignment and spot matching across the gels, (ii) normalization, background adjustment and noise removal, (iii) spot detection, and (iv) quantification by calculation of the spot volumes and statistical analysis to highlighting differentially present proteins. Background cleaning allows the enhancement of the protein signal and distinguishes the noise from a spot. The global background correction consists of subtraction of all pixels below a set threshold of the maximum intensity. For matching, typically a reference gel is chosen and all gels are then automatically matched to the master one. Matching represents the most laborious step since frequent mistakes are made due to gel-to-gel and spot migration variability. Therefore, user intervention is needed to manually correct the software and improve the accuracy in spot matching. The quantification is performed through a summation of the pixel intensities localized within the defined spot area. The softwares use multivariate statistical packages, such as ANOVA (analysis of variance) based on spot size and intensity, spots are then assigned to *P* values, fold changes between groups. Most packages furthermore apply FDR (false discovery rates) or *q*-values to avoid the wrongful assignment of significant changes. PCA (Principle Component Analysis) is also often carried out. These available statistical tests make the 2-DE analyses and quantification more straightforward. However, the challenges associated with computational 2-DE analysis are technical problems such as experimental variation between gels and a high probability of piling several proteins under one spot.

Gel-based proteomics has so far been the main approach used in plant proteomics. 2D-DIGE has been successfully applied to investigate symbiosis- and pathogenesis-related protein in *Medicago truncatula* [16, 17] and to study the impact of abiotic stresses such as drought in oak [18], frost in *Arabidopsis* [19], ozone, and heavy metals in poplar [20–22].

### 2.2. Electrophoretic Separations of Native Proteins

In their endeavour to study the protein complexes of the respiratory chain of mitochondria, Schägger and von Jagow developed a gel-based system able to separate protein complexes involved in oxidative phosphorylation in their native state [23]. This technique enables the separation of protein complexes under native conditions followed by the separation of individual proteins under denaturing conditions, thereby providing insight into the stoichiometry of the complexes. A charge-shifting agent, the dye Coomassie Brilliant Blue G-250, is added to the cathode buffer in order to stick to proteins conferring a uniform electric charge without unfolding the protein structure. Thus, intact protein complexes can be separated on a nondenaturing gradient gel roughly according to their MW, but the size and shape of each complex also influence how far that complex migrates into the gel. The gel lane is then cut out and separated on a second gel and orientated perpendicularly to the first axis of separation. This second dimension, a classic SDS-PAGE, is performed to separate the component proteins of each complex according to their MW. Blue Native-PAGE (BN-PAGE) studies were mainly focused on the analysis of electron transfer chain complexes in plastids and mitochondria; the potential application of this technique in plant proteomics was previously discussed and reviewed [24]. More recently, this strategy was used efficiently to analyze the proteome of wheat chloroplast protein complexes [25]. BN-PAGE was highly linked to membrane proteomics showing a deep interest to improve the hydrophobic proteome coverage of gel-based approaches [26].

BN-PAGE appears to be unsuitable to resolve small protein complexes (*<*100 kDa) due to the small separation distance of the first gel step, nevertheless a protocol for bacteria and eukaryotic cells allowing the identification of complexes in the range of 20–1,300 kDa was recently reported [27]. However, distinct complexes of similar molecular masses may comigrate and the constitutive proteins appear then to be present in the same complex. Despite the trick of the use of a charge-shifting agent, BN-PAGE is difficult to optimize and it is quite common to observe some trailing of the bands, which indicates insufficient protein solubilisation. To improve the resolution, three-dimensional electrophoresis can be performed, combining 2 variants of native electrophoresis in the first and second dimension, and SDS-PAGE in the third dimension [7].

### 2.3. One-Dimensional Gel Electrophoresis (1-DE): The Birth of Proteomics

Soon after its inception, one-dimensional gel electrophoresis (1-DE) became the most popular method for at least two purposes: fast determination of protein MWand assessing the protein purity. Today, this widespread technique is used for many applications: comparison of protein composition of different samples, analysis of the number and size of polypeptide subunits, western blotting coupled to immunodetection, and, of course, as a second dimension in 2-DE maps. 

Taking advantage of both gel-based protein and gel-free peptide separation properties 1-DE is, nowadays, coupled to subsequent analysis in liquid chromatography (LC) prior to MS. After protein separation on SDS gel, the entire gel lane is excised and divided into slices prior to the proteolytic digestion. Afterwards, peptide fractions are subjected to a second separation in LC prior to MS/MS analysis. The main advantages of this technique are the harsh ionic detergent use of the SDS that ensures protein solubility during the size-separation step and the reduced sample complexity prior to LC which renders the chance of identifying low abundant proteins higher. Recently comparisons of 1-DE-LC approach to other fractionation methods (e.g., cation exchange, isoelectric focusing, etc.), at both protein and peptide level, demonstrated its superior performance and higher proteome coverage [28–30]. Thus, by increasing the solubility (the major bottleneck in protein separations) and dwindling the complexity of the system by cutting the protein gel lane, 1-DE coupled to LC/MS analysis represents an attractive technique in proteomics studies. In plants, 1-DE-LC-MS/MS approach has been broadly applied, as an example the study on *M. truncatula* plasma membrane changes in response to arbuscular mycorrhizal symbiosis [31] and on *Arabidopsis thaliana *chloroplast envelope [32]. Lately, this approach has also been used for the compilation of a protein expression map of the *Arabidopsis* root providing the identity and cell type-specific localization of nearly 2,000 proteins [33].

## 3. Proteomics: From Gel-Based to Gel-Free Techniques

*“A la carte proteomics with an emphasis on gel-free techniques” [34]*

Two-dimensional gel electrophoresis is a now a mature and well-established technique, however it suffers from some ongoing concerns regarding quantitative reproducibility and limitations on the ability to study certain classes of proteins. Therefore in recent years, most developmental endeavours have been focused on alternative approaches, such as promising gel-free proteomics. With the appearance of MS-based proteomics, an entirely new toolbox has become available for quantitative analysis. In shotgun proteomics (bottom-up strategy) complex peptide fractions, generated after protein proteolytic digestion, can be resolved using different fractionation strategies, which offer high-throughput analyses of the proteome of an organelle or a cell type and provide a snapshot of the major protein constituents. 

Although these novel approaches were initially pitched as replacements for gel-based methods, they should probably be regarded as complements to rather than replacements of 2-DE. There are many points of comparison and contrast between the standard 2-DE and shotgun analyses, such as sample consumption, depth of proteome coverage, analyses of isoforms and quantitative statistical power. Both platforms have the ability to resolve hundreds to thousands of features, so the choice between the different platforms is often determined by the biological question addressed. Currently there is no single method, which can provide qualitative and quantitative information of all protein components of a complex mixture. Ultimately, these approaches are both of great value to a proteomic study and often provide complementary information for an overall richer analysis. 

## 4. Peptide Fractionation Procedures

*“The introduction of multidimensional peptide resolving techniques is of unquestionable value for the characterization of complex proteomes” [35]*

Since there is no method or instrument that is capable of identifying and quantifying the components of a complex sample in a single-step operation, there is ample evidence that high dimensional fractionation is required for deep exploration of complex proteomes and low abundant proteins. The basic principle of multidimensional fractionation is to separate peptides according to various orthogonal physicochemical properties and/or affinity interactions, resulting in much less complex fractions. There are numerous methodologies of separation available that can be used in tandem to perform a reduction in sample complexity. Each method has its own merits and drawbacks; therefore, the downstream needs of the workflow determine the optimal method for sample analysis. 

### 4.1. Ion-Exchange Chromatography (IEC)

This type of chromatography involves peptide separation according to electric charge. In cation-exchange chromatography (CX), negative functional groups attract positively charged peptides at acidic pH, while in anion-exchange chromatography (AX), positive functional groups have affinity for negatively charged peptides at basic pH. Strong cation-exchange chromatography (SCX) encompasses a strong exchanger group that can be ionised over a broad pH range. For peptide separation using SCX columns, the peptide mixture is loaded under acidic conditions so that the positively charged peptides bind to the column. By increasing the salt concentration, peptides are displaced according to their charge, while by applying a pH gradient, peptides are resolved according to their isoelectric point (pI). Thus, positively charged peptides bind to the SCX column when the actual buffer pH is lower than their pI. 

### 4.2. Reversed-Phase Chromatography (RP)

This most widespread LC-method applied in proteomics allows neutral peptide separation according to their hydrophobicity. The separation is based on the analyte partition coefficient between the polar mobile phase and the hydrophobic (nonpolar) stationary phase. The trapped peptides are then eluted using an organic phase gradient, usually acetonitrile. The ion-pair chromatography relies upon the addition of ionic compounds to the mobile phase to promote the formation of ion pairs with charged analytes. These reagents are comprised of an alkyl chain with an ionisable terminus. The introduction of ion-pair reagents increased the retention of charged analytes and improved peak shapes. Trifluoroacetic acid (TFA) and formic acid (FA) have been extensively used as ion-pairing reagents [35]. 

### 4.3. Two-Dimensional Liquid Chromatography (2D-LC)

Multidimensional analytical methods, having orthogonal separation power, are required to reduce sample complexity and increase the proteome coverage. The separation of peptide mixtures by 2D-LC has been performed using several orthogonal combinations such as AX coupled to RP (AX/RP), size exclusion chromatography coupled to RP (SEC/RP), and affinity chromatography coupled to RP (AFC/RP). In most shotgun proteomic analyses, the second dimension is performed by RP because the mobile phase is compatible with MS [36].

It has been shown that SCX is an excellent match to RP for multidimensional proteomic separations. In offline mode, the eluted fractions of the first dimension (SCX) are collected and then subjected to the second dimension (RP). Online approaches are faster with less sample loss due to the direct coupling of the two dimensions. In multidimensional protein identification technology (MudPIT) the SCX and RP stationary phase are packed together in the same microcapillary column. It was developed in the Yates laboratory and the results showed a high number of protein identifications, including low abundant ones [37]. This technology shows good separation power and presents a prime example of the enhanced proteome coverage in bottom-up proteomic approaches [38]. Several studies employed MudPIT in plant proteomics and its usage in this field was been previously reviewed [39, 40].

### 4.4. OFFGEL Electrophoresis (OGE)

The recently developed OFFGEL fractionator allows liquid-phase peptide IEF. The separation is carried out in a two-phase system with an upper liquid phase, containing carrier ampholytes and buffer-free solution, divided into 12 or 24 compartments and a lower phase, which is the IPG strip [41]. After sample loading into the wells and application of a voltage gradient, peptides migrate through the IPG strip until they reach their pI at a given compartment. After IEF, peptides can be easily recovered in solution for further analysis. OGE has high loading capacity and resolution power [41]. Unlike LC fractionation, OGE provides additional physiochemical information such as peptide pI, which is a highly valuable tool to corroborate MS results, sort false positive rates, and increase the reliability of the identification procedure. While a study comparing MudPIT to OGE fractionation for the high-resolution separation of peptides revealed comparable results using both platforms [42], others showed that the IPG as a first dimension separation strategy is superior to SCX with a salt gradient [43] or pH gradient [44] for the analysis of complex mixtures. In contrast, Yang and coworkers reported that RP-LC offered better resolution and yielded more unique peptide and protein identifications in comparison to OGE in proteomic analysis of differentially expressed proteins in long-term cold storage of potato tubers [45]. During the last few years the use of OGE in plant proteomics has increased. Its application allowed the recovering of wheat soluble proteins extracted from leaves [46]. OGE was furthermore compared to classical IEF on microsomal fractions of 5 plant species. OGE performed slightly better in the identification of proteins with transmembrane domains and significantly increased the number of proteins in the alkaline range [47]. Finally, this technique has also been used on microsomal proteins extracted from *M. truncatula* roots to investigate the iTRAQ labelling effect on peptide isoelectric point and thus their focusing behaviour in OGE [48]. 

The long running time of OGE (which varies from few hours to 2-3 days) in comparison with other offline technique was the main disadvantage associated to this novel technique.

## 5. MS-Based Quantitation

*“Mass spectrometry-based proteomics turns quantitative” [49]*

In the last decade, MS has known a tremendous progress in proteomics and has increasingly established itself as a key tool for the analysis of complex protein samples notably after the availability of protein sequence databases and the development of more sensitive and user-friendly MS equipment [50]. A new toolbox of label-based and label-free quantitative proteomic methods is currently available. “To label or not to label,” to answer this question and select the appropriate quantitative approach some considerations should be taken into account. Different proteomic approaches vary in their sensitivity, and the variability of each method should be defined *a priori* together with the workflow and sample-specific characteristics [51]. The number of biological and technical replicates is also critical, the greater the number of replicates, the more representative the results will be for the general population. Several studies have focused on the comparison of label-based and label-free methods for quantitative proteomics and the results showed that there is no superiority and that the accuracy of the acquired results depends on the experimental set-up [52]. 

## 6. Overview of Label-Based Proteomic Approaches

*“Stable isotope methods for high-precision proteomics” [53]*

The labelling methods for relative quantification studies can be classified into two main groups: chemical isotope tags and metabolic labelling. These approaches are based on the fact that both labelled and unlabelled peptides exhibit the same chromatographic and ionisation properties but can be distinguished from each other by a mass-shift signature. In metabolic labelling, the label is introduced to the whole cell organism through the growth medium, while in chemical labelling, proteins or peptides are tagged through a chemical reaction [3].

### 6.1. Chemical Labelling

#### 6.1.1. Proteolytic Labelling

^18^O stable-isotope labelling is a simple, fast, and reliable method that takes place during proteolytic digestion in presence of heavy water (H_2_^18^O) [54]. Samples undergo enzymatic digestion either in presence of H_2_^16^O (unlabelled sample) or H_2_^18^O (labelled sample). The natural catalytic activity of serine proteases (e.g., trypsin, Lys-C, and Arg-C) can exchange both C-terminal oxygen atoms with a “heavy” ^18^O from water in the surrounding solution. The first ^18^O atom is introduced upon the cleavage of the peptidic amide bond, while the second ^18^O atom is introduced when the cleaved peptide is bound to the enzyme as a reaction-mechanism intermediate (Table 1). The resulting peptides, 2 or 4 Da heavier than their unlabelled counterparts, are pooled with the unlabelled peptide mixture and peak intensities of the isotopic envelopes are compared, which can be resolved in medium-high resolution mass spectrometers [55]. Trypsin-catalyzed ^18^O isotopic labelling has not often been used in plant proteomics and only one application was found (Table 2). Nelson and coauthors has used ^18^O isotopic labelling for relative quantification of the degree of enrichment of *Arabidopsis* plasma membrane proteins [56]. The main drawback of this technique, despite optimization by Staes et al. [57], is that the exchange reaction is rarely complete for all peptides, resulting in a complex isotopic pattern due to the overlap of the unlabeled and singly and doubly labelled peptides. 

#### 6.1.2. Isotope-Coded Affinity Tags (ICAT)

One of the first labels used for differential isotope labelling consists of three functional elements: a specific chemical reactive group that binds to sulfhydryl groups of cysteinyl residues, an isotopically coded linker with light or heavy isotopes, and a biotin tag for affinity purification (Table 1) [58]. The proteins containing cysteine residues are labelled either with light or heavy isotopes, where the latter form has eight ^13^C atoms. Afterwards, light- and heavy-labelled samples are pooled and proteolytically cleaved. Subsequently, the complexity of the sample is reduced prior to MS analysis through the purification of tagged cysteine-containing peptides by affinity chromatography using biotin-avidin affinity columns. Peptide pairs with 8 Da mass-shifts are detected in MS scans and their ion intensities are compared for relative quantitation. ICAT labelling takes place at the protein level allowing samples to be pooled prior to protease treatment, thus eliminating vial-to-vial variations. However, cysteine is not very abundant and approximately one in seven proteins do not contain this amino acid, greatly reducing the completeness of the study [59].

In plants, Dunkley and coworkers have studied the localization of organelle proteins by isotope tagging (LOPIT) to discriminate endoplasmic reticulum, Golgi, plasma membrane, and mitochondria or plastid proteins in *Arabidopsis*. This technique involves partial separation of the organelles by density gradient centrifugation followed by the analysis of protein distributions in the gradient by ICAT and MS [60]. Taking advantage of the ICAT labelling specificity to cysteinyl groups, this approach was used to study the redox-status of proteins allowing a quantitative analysis of the redox proteome and ozone stress in plants [61–64]. To increase the functional information about S-nitrosylation sites in plants, Fares and colleagues combined both “biotin-switch” method (BSM) and ICAT labelling and succeeded in identifying 53 endogenous nitrosocysteines in *Arabidopsis* cells [65]. ICAT was also used to identify wheat seed proteins and to understand their interactions and expression in relation to chromosome deletion, which were reported to be difficult by 2-DE due to co-synthesis of proteins by genes from three genomes, A, B and D [66]. A cross-comparison of gel-based and -free quantitative methods (2-DE, ICAT, and label-free) was performed by analysing the differential accumulation of maize chloroplast proteins in bundle sheath versus mesophyll cells. Among the 125 chloroplast proteins quantified in the 3 methods, only 20 proteins were quantified in common, demonstrating the complementary nature of these quantitative approaches [67]. More applications of ICAT quantitative approach in plant proteomics are listed in Table 2.

#### 6.1.3. Isotope-Coded Protein Labelling (ICPL)

This approach termed ICPL is based on isotopic labelling of all free amino groups in proteins. Two protein mixtures are reduced and alkylated to ensure easier access to free amino groups that are subsequently derivatised with the deuterium-free (light) or 4 deuterium containing (heavy) form, respectively (Table 1). Light- and heavy-labelled samples are then mixed, fractionated, and digested prior to high throughput MS analysis. Since peptides of identical sequence derived from the two differentially labelled protein samples differ in mass (4 Da), they appear as doublets in the acquired MS spectra. From the ratios of the ion intensities of these sister peptide pairs, the relative abundance of their parent proteins in the original samples can be determined [68]. Recently, a detailed experimental protocol called postdigest ICPL was published highlighting a better protein identification and quantification [69, 70] and when compared to iTRAQ, both techniques have shown comparable number of identified and quantified proteins in the endosperm of castor bean seeds at three developmental stages [71]. So far, the latter study is the unique reported quantitative proteomic investigation on plants using ICPL (Table 2). The main drawback of this method is the isotopic effect of deuterated tags that interferes with retention time of the labelled peptides during LC [72]. 

#### 6.1.4. Isobaric Tags for Relative and Absolute Quantification (iTRAQ)

Unlike ICAT and ICPL, iTRAQ tags are isobarics and primarily designed for the labelling of peptides rather than proteins. The overall molecule mass is kept constant at 145 Da and 304 Da for iTRAQ-4plex and -8plex, respectively. The structure of the iTRAQ-8plex balancer group has not been published while the iTRAQ-4plex molecule consists of a reporter group (based on N-methylpiperazine), a mass balance group (carbonyl), and a peptide reactive group (NHS ester) (Table 1) [73]. The iTRAQ reagents label peptide N-termini and *ε*-amino groups of lysine side chains and allow comparison of up to eight samples in the same experiment. Another difference from the pre-cited methods is that the quantification occurs in MS/MS scans after peptide fragmentation. In fact, iTRAQ-labelled peptides appear as a single unresolved precursor at the same m/z in the MS spectrum. Upon peptide fragmentation, the iTRAQ labels fragment to produce reporter ions in a “silent region,” usually unpopulated, at low m/z range (e.g., 114–121). Measurements of the reporter ion intensities enable relative quantification of the peptide in each sample.

This method has quickly gained popularity in proteomics and benefits from increased MS sensitivity compared to for instance ICAT due to the contribution of all samples to the precursor ion signal. The iTRAQ reagent was furthermore reported to increase the number of lysine-terminated tryptic peptides identified by database searches to equivalence with arginine-terminated peptides [73]. Ow and coauthors evaluated iTRAQ relevance, accuracy, and precision for biological interpretation and entitled their verdict “the good, the bad and the ugly” of iTRAQ quantitation [74]. “The good” is the potential of iTRAQ to provide accurate quantification spanning two orders of magnitude. However, that potential is limited by two factors: isotopic impurities “the bad”, and peptide cofragmentation (inadvertently selecting two or more closely spaced peptides for MS/MS instead of one) “the ugly” [75]. In the same study, a putative contamination of the reporter ion region with the second isotope of the phenylalanine immonium ion on the 121 m/z peak, which can interfere with peptide quantification was mentioned [74]. 

The iTRAQ has shown a high utility in large-scale quantitative proteomics (Table 2) to study plant responses to pathogens: *Pseudomonas syringae* in *Arabidopsis* [76], *Lobesia botrana* and *Erysiphe necator* in grape [77, 78], *Huanglongbing* in sweet orange [79], *Fusarium graminearum* in maize [80]. Quantitative shotgun proteomic approaches using iTRAQ were furthermore used for characterizing the differential phosphorylation of Arabidopsis in response to microbial elicitation [81] and the study of protein degradation in chloroplasts [82]. The potency of iTRAQ was used for better understanding mechanisms of plant tolerance to boron in barley [83], cadmium in barley [84] and *Brassica juncea* [85], and cold in potato and rice [45, 86]. An example of iTRAQ application in plant membrane proteomics is the study of differentiated state of bundle sheath and mesophyll chloroplast thylakoid and envelope membrane proteomes in maize [87].

#### 6.1.5. Tandem Mass Tag (TMT)

A novel MS/MS-based quantitative method using isotopomer labels, similar to iTRAQ, and referred to as “tandem mass tags” (TMT) was recently developed (Table 1) [88]. Both techniques share several common features. (i) These reagents employ *N*-hydroxy-succinimide (NHS) chemistry that permits specific tagging of primary amino groups. (ii) They were designed to allow multiplexing of several samples by chemical derivatization with different forms of the same isobaric tag that appear as a single peak in full MS scans. (iii) The release of “daughter ions” in MS/MS analysis (between 126 and 131 Da for TMT) that can be used for relative quantification. The cysteine-reactive TMT (cysTMT) reagents enable selective labelling and relative quantitation of cysteine-containing peptides from up to six biological samples. This technique has been used for the redox proteomic analysis of the tomato leaves in response to the pathogen *P. syringae* pv. tomato strain DC3000 (Table 2) [89]. Aside from this study, TMT labelling approach has so far not been fully exploited for the analysis of plant proteomes.

A study comparing TMT and iTRAQ showed that the performance of both techniques was similar in terms of quantitative precision and accuracy, however the number of identified peptides and proteins was higher with iTRAQ 4-plex compared to TMT 6-plex [116]. 

### 6.2. Metabolic Labelling

Although chemical labelling presents a wide range of approaches for quantitative proteomics, this group of techniques suffers from sample variability and induces a technical bias since the labelling occurs after the protein extraction or even after proteolytic digestion. In addition, the high cost of these reagents can be a limiting factor for large-scale experiments. Therefore metabolic labelling, which allows protein labelling at the time of protein synthesis, presents a valuable alternative strategy for quantitative proteomics.

#### 6.2.1. Stable Isotopic Labelling with Amino Acids in Cell Culture (SILAC)

*In vivo* metabolic labelling, in which two populations of cells are cultured either in a medium containing a “light” (unlabelled) amino acid or encompassing a “heavy” (labelled), one typically arginine or lysine labelled with ^13^C and/or ^15^N are used [117]. The mass shift induced by the incorporation of the heavy amino acid into a peptide, is known and allows comparison between a peptide in both samples (e.g., 6 Da in the case of ^13^C_6_-Lys, Table 1). Samples are then combined prior to protein extraction, which minimizes technical variation arising during sample processing. In MS spectra, each peptide appears as a pair and the ratio of peak intensities yields the protein abundance in the sample since the light and heavy amino acids are chemically identical and only isotopically distinguished. 

Although probably the most general and global labelling strategy, SILAC appears less suited for quantitative proteomic studies in plants. Being autotrophic organisms, plants are metabolic specialists capable of synthesising all amino acids from inorganic nitrogen and, therefore, have lower incorporation efficiency of the exogenously supplied labelled amino acids. The labelling efficiency achieved using exogenous amino acid feeding of *Arabidopsis* cell cultures has been found to average only 70–80% [118]. Considering these limitations and the high cost of isotopically labelled amino acids, SILAC appears likely to be inadequate for quantitative proteomics studies in plants; albeit it seems less restricted to study algae such as *Chlamydomonas reinhardtii* and *Ostreococcus tauri* (Table 2) [92–94]. 

#### 6.2.2. ^14^N/^15^N Labelling

In this method, the label is introduced to the whole cell or organism through the growth medium. Samples can easily be labelled metabolically via growth media containing ^15^N-labelled inorganic salts, typically K^15^NO_3_ [95]. The quantification process is based on the intensity of extracted ion chromatograms of survey scans containing the pair of labelled (^15^N, heavy) and unlabelled (^14^N, light) peptide isoforms.

Unlike SILAC, this approach achieved more than 98% incorporation in both plants [95] and cell cultures [96], and is more efficient at allowing large-scale quantitative analysis. The tradeoff is that all amino acids will incorporate the label, thus the mass shift will be peptide-sequence dependent. Metabolic ^15^N-labelling is becoming the method of choice for quantitative proteomics in plant studies (Table 2). It was used to study plant membrane proteome changes in response to cadmium, and cryptogenin elicitor in *Arabidopsis* and tobacco cells, respectively [97, 98]. Such a quantitative proteomic strategy was applied in quantitative phosphoproteomics to study differentiated proteins in response to fungal or microbial elicitors in *Arabidopsis* cells [99]. Moreover, other metabolic labelling strategies have been developed such as hydroponic isotope labelling of entire plants (HILEP) which has proven to be very efficient and robust method to completely label the whole mature plants. Nearly 100% of ^15^N-labelling efficiency was achieved in *Arabidopsis* plants by growing them in hydroponic media containing 2.5 mM ^15^N potassium nitrate and 0.5 mM ^15^N ammonium nitrate [100, 119]. A similar quantitative proteomic method, SILIP (Stable Isotope Labelling InPlanta), was developed for labelling tomato plants growing in sand in a greenhouse environment [101]. An alternative strategy for quantitative proteomics that relies upon the subtle changes in isotopic envelope shape resulting from partial metabolic labelling to compare relative abundances of labelled and unlabelled peptides has been developed in *Arabidopsis*. Both partial and full labelling have been proven to be comparable with respect to dynamic range, accuracy, and reproducibility, and both are suitable for quantitative proteomics characterization [102].

## 7. Label-Free Quantitative Proteomics

*“Comparative LC-MS: a landscape of peaks and valleys” [120]*

*“Less label, more free” [121]*

Quantitative proteomics based on stable isotope-coding strategies often require expensive labelling reagents, high amount of starting samples, multiple sample preparation steps resulting in considerable sample loss and reduced detection sensitivity. Label-free LC/MS methods represent attractive alternatives [122] since they are amenable to all type of biological samples, are simple, reproducible, cost effective, and less prone to errors and side reactions related to the labelling process.

Given the fact that, theoretically, the peak intensity of any ion should be proportional to its abundance the ion signals in MS have been used, for decades, as a quantification technique for small molecules in analytical chemistry. However, technical variation, at both LC and ionization levels, might render comparisons of peak intensities between experiments unreliable. The recent advances in LC/MS approaches allowed circumvention of the looming replicate biases and recently the observation of a correlation between protein abundance and peak areas [123, 124] or number of MS/MS spectra [125] has widened the choice of analytical procedure in the field of quantitative proteomics. The general framework of label-free quantification can be summarised as follows: for the two samples that need to be compared quantitatively, the LC-MS/MS experiment is first performed for both samples separately, and precursor ion m/z and retention time (*Rt*) file is generated for all MS/MS spectra of each identified protein, creating a 2D map (m/z, *Rt*) allowing peptide match in several samples. 

Depending on the MS acquisition mode, two analytical methods can be distinguished: the data-dependent analysis (DDA) and the data-independent analysis (DIA). DDA involves acquisition of a MS survey scan followed, for an allotted period of time, by precursor ion selection based on its intensity for subsequent fragmentation [126]. In this approach, quantification can be achieved using DDA-based spectral counting or spectral peak intensities. Venable and coauthors described DIA in which no parent ion is preselected; the instrument constantly operates in MS/MS mode and data acquisition of all charge states of eluted peptides is performed by rapid switching of the collision energy between low and high-energy states [127]. 

### 7.1. Spectral Counting

Spectral counting or peptide identification frequency is becoming popular in label-free quantification due to its simple procedure that does not require chromatographic peak integration or retention time alignment. It is based on the rationale that peptides from more abundant proteins will be more selected for fragmentation and will thus produce a higher number of MS/MS spectra. Thus, the number of MS/MS scans is tabulated and the protein abundance is inferred from the total number of MS/MS spectra that match peptides from the protein [125]. The ability to accurately quantify proteins by spectral counting largely depends on the number of spectra obtained and the coverage of sampling. The relative difference in protein abundance is estimated by calculating the protein abundance index (PAI), which corresponds to the number of observed peptides in the experiment divided by the number of theoretical tryptic peptides for each protein within a given mass range of the employed mass spectrometer [128]. The exponential form of PAI minus one (10^PAI^-1), exponentially modified protein abundance index (emPAI) [129], takes into account the fact that generally more peptides are detected for larger proteins and is directly proportional to the protein content in the sample. The absolute protein expression (APEX) index, a very similar approach to emPAI, is a derived measurement of protein abundance in a given sample based on the analytical features in mass spectrometric analysis [130]. It has been used to generate a protein abundance map of the *Arabidopsis* proteome [103] and to determine the abundance of stromal proteins in *A. thaliana* chloroplast [104]. Spectral counting based quantitative proteomics has been widely used in the field of plant proteomics (Table 2). The accuracy and reliability of label-free spectral counting in the relative quantitative analysis of soybean leaf proteome was evaluated by comparing nine technical replicates [105]. Gammulla and coauthors quantified and identified temperature stress responsive proteins in rice leaves by calculating the NSAF (Normalized Spectral Abundance Factor), which is given by the total number of MS/MS spectra (SpC) identifying a protein, divided by the protein's length (L), divided by the sum of SpC/L for all proteins in the experiment [106, 131]. Spectrum counting has been used to study drought stress response in root nodules of *M. truncatula* [132] and in large-scale plant proteomics in response to pathogen infection in bean (*Phaseolus vulgaris*) [133]. 

### 7.2. Spectral Peak Intensities

Other label-free methods use the signal intensities of individual peptides rather than the spectral counts to compare the relative abundance of proteins between samples [134]. It is based on the principle that the relative abundance of the same peptide in different samples can be estimated by the precursor ion signal intensity across consecutive LC/MS runs, given that the measurements are performed under identical conditions. In contrast to differential labelling, every biological specimen needs to be measured separately in a label-free experiment. Typically, peptide signals are detected at the MS level, their patterns are then tracked across the retention time dimension and used to reconstruct a chromatographic elution profile of the monoisotopic peptide mass. The total ion current of the peptide signal is then integrated and the measurement of the chromatographic peak areas is used as a quantitative measurement for the original peptide concentration. Profiling methods based on ion intensity were applied to define the sucrose-induced phosphorylation changes in *Arabidopsis *plasma membrane proteins [107]. It has been furthermore used to detect twelve phosphopeptides from 50 identified phosphoproteins in different amounts during the hypersensitive response in tomato plants [108]. Moreover, the ion intensity method was used as strategic track to study soybean plasma membrane proteins following 24 h flooding and 48 h osmotic stress (Table 2) [109, 135]. 

Spectral counting and spectral peak intensities were compared and results obtained from both methods are generally in good accordance [110, 136] with spectral counting covering a slightly higher dynamic range and measurements of ion abundance being more accurate for the identification of protein ratios [136]. Both techniques have also been used to investigate the major allergens in transgenic peanut lines [111].

Unlike labelling methods, in which quantitative analyses are limited to the tagged peptides, label-free approaches offer the quantitative comparison of all peptide constituents of the sample. However, they are more susceptible to errors due to parallel sample processing and thus suffer from increased analytical variability. Therefore, label-free methods are very replicate dependent. To be statistically significant, chromatographic separation reproducibility must be very high. The high-resolution power of MS, high scanning rates, high accurate mass measurements, and exact chromatogram alignment are prerequisite for the success of this quantitative technique [134, 137]. The extensive workflow ranging from peptide detection, alignment, normalization, identification, quantitative comparisons, and statistical analysis has triggered the development of several sophisticated software algorithms. 

### 7.3. Data-Independent Analysis (DIA)

LC/MS^E^, a quantitative comparison of ions emanating from identically prepared control and experimental samples, was developed by using a reproducible chromatographic separation system along with the high mass resolution and mass accuracy of an orthogonal time-of-flight mass spectrometer [134]. In this method, the instrument alternates between low and high collision energies in MS analysis. While the low collision energy scan mode leads to the determination of accurate precursor ion masses, the high-energy scan mode (MS^E^) generates accurate peptide fragmentation data [26]. The use of multiplex parallel fragmentation of LC/MS^E^ yields uniformly product ion information of all peptides across their entire chromatographic peaks [134], which provides continuous MS data throughout the entire acquisition. Product ions are time-aligned and correlated to precursor ions to generate a list of exact mass retention time (EMRT) signatures [134]. The integrated peak areas of EMRT are compared across different biological replicates to determine the differences in protein abundances. 

The LC/MS^E^ approach is well suited for relative and absolute quantification [112] and it was shown to increase the signal-to-noise ratio by a factor 3–5 and could identify peptides undetected in a parent ion scan [138]. This recent achievement in MS-based proteomics has provided a basis to qualitatively and quantitatively assess the transition from dark to light of maize seedlings [113] and to study the salicylic acid-induced changes in the *Arabidopsis *and* Apium graveolens* secretome [114, 139]. MS^E^ has also been implemented to study the changes in barley protein expression in response to UV-B treatment (Table 2) [115]. 

## 8. Conclusion

Proteomics, the promising new “omics,” has become an important complementary tool to genomics providing novel information and greater insight into plant biology. The application of gel-based and -free proteomics methods to study plant physiology has strongly increased in recent years. Here, a broad perspective is offered on the available techniques.

So far, most quantitative plant proteomics was performed on *Arabidopsis thaliana*, the model plant due to various traits including its small (and annotated) genome size (125 MBp), short generation time, high transformation efficiency, and the large panel of available mutants. The completion of more plant genome sequencing projects such as rice, barley, tomato and *M. truncatula* and will permit the proteome probing of these plant systems. In the meantime, extensive EST databases for numerous important crop plants represent alternative sources of sequence information to the full genome sequences. Moreover, with the technical maturity attained in MS and protein/peptide fractionation tools, comparative plant proteomics will move out of the beginner realm and emerge as high valuable discipline to enhance the comprehension of plant systems, their subcellular membranes and organelles. It is worth noting that combining multiple quantitative proteomic techniques is highly beneficial, as these approaches yield complementary datasets which improve the understanding of biological issues and provide in-depth characterization of proteins with respect to their abundance. These technical advancements coupled to well-designed experiments will significantly reveal the protein function in plant growth and development and provide a wealth of information on plant proteome changes occurring in response to external stimuli, biotic, and abiotic stresses. 

## Figures and Tables

**Table 1:** The various available isotopic labels, their sites of labeling, and structures.

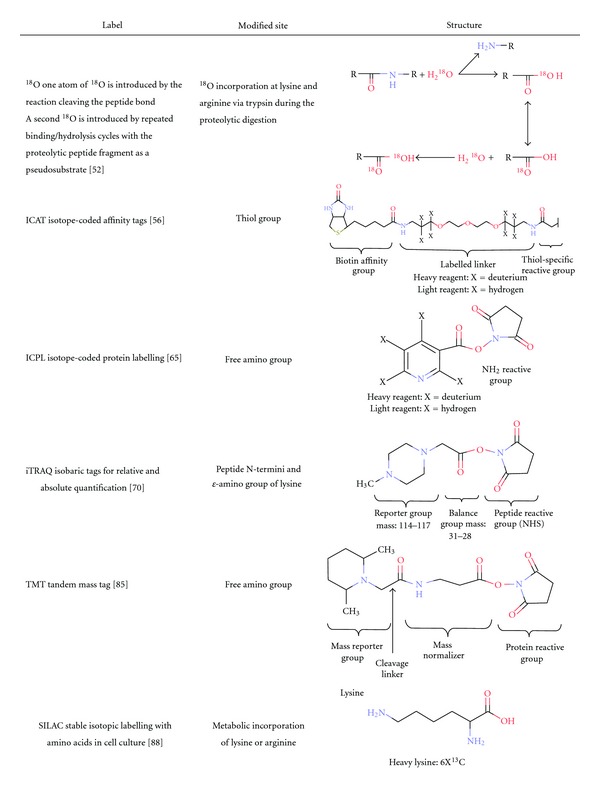

**Table 2:** An overview of the latest MS-based quantitative proteomic studies on plant systems. The table shows the implemented quantitative approaches, plant species, biological questions, and reference of the corresponding paper.

Quantitative approach	Plant	Biological study	Authors
^ 18^O labelling	*Arabidopsis thaliana *	Quantification of the degree of plasma membrane protein enrichment	[56]
ICAT	*Arabidopsis thaliana *	Localization of integral membrane proteins by using the localization of organelle proteins by isotope tagging (LOPIT)	[60]
ICAT	*Hordeum vulgare *	Identification of specific disulfide targets of barley thioredoxin in proteins released from barley aleurone layers	[63]
ICAT	*Arabidopsis thaliana *	Understanding of AtMPK6 role in transducing ozone-derived signals	[64]
ICAT	*Arabidopsis thaliana *	Functional information about S-nitrosylation sites in plants	[65]
ICAT	*Triticum aestivum *	Identification of wheat seed proteins and their related expression to chromosome deletion	[66]
ICAT, 2-DE, label-free	*Zea mays *	Quantitative comparative proteome analysis of purified mesophyll and bundle sheath chloroplast stroma in maize	[67]
ICAT	*Oryza sativa *	Protein profiling of uninucleate stage rice anther and identification of the CMS-HL-related proteins	[90]
ICAT	*Arabidopsis thaliana *	ProCoDeS (proteomic complex detection using sedimentation) for profiling the sedimentation of a large number of proteins	[91]
ICPL, iTRAQ	*Ricinus communis *	Quantitative proteomic comparison of ICPL versus iTRAQ on *ricinus communis* seeds	[71]
iTRAQ	*Solanum tuberosum *	Comparative proteomic approach of potato tubers after 0 and 5 months of storage at 5°C	[45]
iTRAQ	*Arabidopsis thaliana *	Quantitative study of the secreted proteins from *Arabidopsis* cells in response to *Pseudomonas syringae *	[76]
iTRAQ	*Vitis vinifera *	Comparative proteomic study of dynamic changes in control and infected *Vitis vinifera *	[77]
iTRAQ	*Vitis vinifera *	Comparative analysis of differentially expressed proteins in *Erysiphe necator* infected grape	[78]
iTRAQ	*Citrus sinensis *	Comparative proteomic approach of the pathogenic process of HLB in affected sweet orange leaves	[79]
iTRAQ	*Zea mays *	Proteomic approach of two maize inbreds in the early infection by *Fusarium graminearum *	[80]
iTRAQ	*Arabidopsis thaliana *	Changes tack of the Arabidopsis phosphoproteome during the defence response to *Pseudomonas syringae *	[81]
iTRAQ	*Arabidopsis thaliana *	Investigation of the proteomic changes in the chloroplasts of clpr2-1	[82]
iTRAQ	*Hordeum vulgare *	Comparative proteomic study of boron-tolerant and -intolerant barley	[83]
iTRAQ	*Hordeum vulgare *	Quantitative proteomic approach to unravel the contribution of vacuolar transporters to Cd^2+^ detoxification	[84]
iTRAQ, 2D-DIGE	*Brassica juncea *	Quantitative proteomic approaches to understand the effect of cadmium on *Brassica juncea* roots	[85]
iTRAQ, label-free	*Oryza sativa *	Quantitative proteomic response of rice seedling to 48, 72, and 96 h of cold stress	[86]
iTRAQ, BN-PAGE, label-free	*Zea mays *	Comparative analysis of protein abundance in chloroplast thylakoid and envelope membrane proteomes in maize	[87]
Cys-TMT	*Solanum lycopersicum *	Study of the redox proteomic analysis of the *Pseudomonas syringae* tomato DC3000 treated tomato leaves	[89]
SILAC	*Chlamydomonas reinhardtii *	Dynamic changes of proteome turnover under salt stress	[92]
SILAC	*Ostreococcus tauri *	Quantitative proteomics on synthesis and degradation rate constants of individual proteins in autotrophic organisms	[93]
SILAC	*Chlamydomonas reinhardtii *	Comparative proteomics on the iron deficiency impact in *Chlamydomonas reinhardtii *	[94]
^ 14^N/^15^N labelling	*Solanum tuberosum *	Effectiveness of fully label a plant with ^15^N isotopes	[95]
^ 14^N/^15^N labelling	*Arabidopsis thaliana *	Demonstration of plant ^15^N labelling as a powerful comparative quantitative proteomic approach	[96]
^ 14^N/^15^N labelling	*Arabidopsis thaliana *	Comparative analysis of *Arabidopsis* cells following a cadmium exposure	[97]
^ 14^N/^15^N labelling	*Nicotiana tabacum *	Quantitative proteomic approach of the detergent-resistant membranes of tobacco cells in response to cryptogenin	[98]
^ 14^N/^15^N labelling	*Arabidopsis thaliana *	Quantitative approach of phosphorylated sites in signaling and protein response in flg22 or xylanase *Arabidopsis*-treated cells	[99]
HILEP	*Arabidopsis thaliana *	Demonstration of HILEP suitability for relative plant quantitative proteomic subjected to oxidative stress	[100]
SILIP	*Solanum lycopersicum *	SILIP development for homogeneously ^15^N incorporation within the whole plant proteome	[101]
^ 14^N/^15^N labelling	*Arabidopsis thaliana *	Investigation of both partial and full ^15^N labelling effect on quantitative analysis in a complex mixture	[102]
Spectral counting	*Arabidopsis thaliana *	Proteome map of *Arabidopsis * *thaliana *	[103]
Spectral counting	*Arabidopsis thaliana *	Comprehensive *Arabidopsis* chloroplast proteome analysis	[104]
Spectral counting	*Glycine max *	Evaluation of the suitability of spectral counting to quantitative soybean proteome study	[105]
Spectral counting	*Oryza sativa *	Differential proteomic response of rice leaves exposed to high- and low-temperature stress	[106]
Peak ion intensity	*Arabidopsis thaliana *	Sucrose-induced phosphorylation changes of plasma membrane proteins in *Arabidopsis *	[107]
Peak ion intensity	*Solanum lycopersicum *	Quantitative proteomics of phosphoproteins in tomato hypersensitive response	[108]
Peak ion intensity, 2-DE	*Glycine max *	Investigation of the soybean plasma membrane function in response to flooding stress	[109]
Spectral counting + peak ion intensity	*Medicago truncatula *	Comparison of two label-free quantitative approaches on nodule protein extracts from *Medicago truncatula *	[110]
Spectral counting + peak ion intensity	*Arachis hypogaea *	Investigation of major allergens in transgenic peanut lines	[111]
MS^E^	*Apium graveolens *	Analysis of the *Apium graveolens* protein response to salicylic acid	[112]
MS^E^	*Zea mays *	Proteomic approach assessment of the transition from dark to light in maize seedlings	[113]
MS^E^	*Arabidopsis thaliana *	Proteomic changes in the cell wall proteome in response to salicylic acid	[114]
MS^E^	*Hordeum vulgare *	Study of the UV-B irradiation effect on the barley proteome	[115]
